# Evaluating sex hormones and cytokine profile in Egyptian females with relapsing-remitting multiple sclerosis

**DOI:** 10.1186/s41983-018-0030-2

**Published:** 2018-11-03

**Authors:** Forayssa M. Talaat, Noha T. Abokrysha, Dalia M. Labib, Engy El Khateeb, Ghada Hatem Abd El Aziz

**Affiliations:** 1 Neurology Department, Faculty of Medicine, Cairo University, Giza, Egypt; 2 Clinical Pathology Department, Faculty of Medicine, Cairo University, Giza, Egypt

**Keywords:** Relapsing-remitting multiple sclerosis, Estrogen, Testosterone, Interleukin 10, Interleukin 4, Tumor necrosis factor alpha

## Abstract

**Background:**

Sexual dimorphism shown in multiple sclerosis suggests an interaction between immune system and sex hormones. The objective of this study is to determine the hormonal profile and serum cytokine levels in Egyptian female patients with relapsing-remitting MS (RRMS) compared with healthy controls and their associations with disease disability.

**Methods:**

This study was conducted on 40 female patients with RRMS and 20 age-matched controls subjected to measurements of the hormonal profile (estrogen, testosterone) and cytokine levels (interleukin 10 and 4 and tumor necrosis factor alpha) and disability assessment using Expanded Disability Status Scale (EDSS).

**Results:**

Levels of estrogen, testosterone, interleukin 10 and 4 (IL-10 and IL-4), and tumor necrosis factor alpha (TNF-α) were higher in patients compared to control with no statistically significant difference. Estrogen levels were positively correlated with interleukin 10 and interleukin 4 levels and negatively correlated with tumor necrosis factor alpha (TNF-α), but there was no statistically significant correlation between hormonal profile or cytokine profile (IL-10, IL-4, and TNF-α) and EDSS.

**Conclusions:**

It is suggested that estrogen has an anti-inflammatory effect on cytokine milieu; therefore, it can be tried as a treatment option in multiple sclerosis.

## Background

Multiple sclerosis (MS) is an autoimmune demyelinating disease of the central nervous system (CNS), having two pathogenic components, inflammation and neurodegeneration [1]. The female predominance especially during reproductive ages in multiple sclerosis disease suggests that sex hormones interact with the immune system [2].

The exacerbations usually occur immediately premenstrual, when estrogen levels are low [3]. Also, the relapse of disease symptoms lowers during the last 3 months of pregnancy and again increases after delivery [4]. Furthermore, Tomassini and colleagues reported worsening of MS symptoms in about 50% of patients at menopause [5]. Alonso and colleagues reported that subjects receiving contraceptive therapy might be associated with a short-term reduction in the risk of developing MS. These changes are said to be due to immunological and hormonal changes [6]. Sex hormones appear to be involved in regulating the balance of cytokine secretion by T helper 1 and 2 (Th1, Th2), T regulatory (T reg cells), and antigen-presenting cells (APCs). The production of cytokines is the target of sex hormones in the regulation of the clinical manifestation of MS.

The objective of our study is to determine the hormonal profile and serum cytokine in Egyptian female patients with relapsing-remitting MS (RRMS) compared with healthy controls and their associations with disease disability.

## Subjects and methods

### Subjects

This was a cross-sectional study conducted on 40 Egyptian female patients with relapsing-remitting multiple sclerosis diagnosed according to the McDonald criteria 2010 with regular menstrual cycles, and all were in remission. They were recruited from the MS unit of the Neurology Department in the Kasr Al-Ainy Hospital, Cairo University, from January 2015 to November 2016. The age of patients ranged from 18 to 38 years with mean age of 30.22 ± 4.68.

Excluded patients presented with progressive multiple sclerosis, history of steroid intake, or immunosuppressive treatment in the past 2 months prior to involvement in the study or history of immunomodulatory treatment and conditions known to be associated with changed cytokine level as post-traumatic head injury, post-stroke epilepsy, Alzheimer’s disease, Parkinson’s disease, cerebral palsy, impaired cognition, malignant brain tumors, hypothyroidism, liver or renal disease, immune-mediated diseases, or inflammatory diseases and patients on histamine receptor H2 antagonists or anti-inflammatory drugs. We also excluded patients on hormonal contraceptive or with history of hormone replacement therapy and with polycystic ovaries, pituitary adenoma, or hypophysectomy.

Twenty healthy female (age-matched) subjects with regular menstrual cycles, with ages ranging from 25 to 38 years and mean age of 30.67 ± 3.71, served as a control group.

Informed consents were taken from all subjects, and the study was approved by the local ethics committee.

### Methods

All subjects were subjected to the following: full history taking including the history of hormonal contraception or replacement therapy and history of previous treatments with immunosuppressive or immunomodulatory medications to verify exclusion criteria; complete general and neurological examinations; and evaluation of disability using the Expanded Disability Status Scale (EDSS).

Laboratory investigations were done to detect serum levels of anti-inflammatory cytokine (interleukin 10 (IL-10) and interleukin 4 (IL-4)), pro-inflammatory cytokine (tumor necrosis factor alpha (TNF-α)), and hormonal profile (estrogen and testosterone).

Peripheral blood samples were withdrawn from all subjects in fasting condition and without anticoagulant. Samples were withdrawn during the follicular phase (day 3 to day 9 of the menstrual cycle) and measured using a sandwich enzyme-linked immunosorbent assay (ELISA).

Regarding interleukin-10, interleukin 4, and tumor necrosis factor alpha assay (supplied by eBioscience, San Diego, USA), samples were coagulated at room temperature, then centrifuged at approximately 1000×*g* for 10 min and stored at − 20 °C. An anti-human (IL-10, IL-4, or TNF alpha) coating antibody was adsorbed onto microwells, human (IL-10, IL-4, or TNF alpha) in the sample or standard binds to antibodies adsorbed to the microwell. Following incubation, unbound biotin-conjugated anti-human (IL-10, IL-4, or TNF alpha) antibody was removed during the wash step. Streptavidin-horseradish peroxidase (HRP) was added and binds to the biotin-conjugated anti-human (IL-10, IL-4, or TNF alpha) antibody. Following incubation, unbound streptavidin-HRP was removed during the wash step and substrate solution reactive with HRP is added to the wells. A colored product was formed in proportion to the amount of human (IL-10, IL-4, or TNF alpha) present in the sample or standard. The reaction is terminated by the addition of acid, and absorbance is measured at 450 nm. A standard curve was prepared from seven human (IL-10, IL-4, or TNF alpha) standard dilutions and human (IL-10, IL-4, or TNF alpha) sample concentration determined.

Regarding estrogen and testosterone assay (supplied by ALPCO Diagnostics, New Hampshire, USA and Diagnostic Biochem Canada Inc., London, Ontario, Canada, respectively), samples were left to coagulate at room temperature then centrifuged and stored at − 10 °C. These assays were based on a standard sandwich enzyme-linked immunosorbent assay technology. Fifty microliters of each calibrator, control, and specimen sample was pipetted into correspondingly labeled wells in duplicate. One hundred microliters of the conjugate working solution was pipetted into each well and incubated on a plate shaker (200 rpm) for 1 h at room temperature. The wells were washed three times with 300 μl of diluted wash buffer per well, and the plate was tapped firmly against absorbent paper to ensure it is dry. One hundred and fifty microliters of tetramethylbenzidine substrate was pipetted into each well at timed intervals and incubated on a plate shaker for 10–15 min at room temperature, then, 50 μl of stopping solution was pipetted into each well at timed intervals, and finally, the plate on the microwell plate reader was read at 450 nm within 20 min after the addition of stopping solution.

The expected normal value for testosterone in females is 0.2–1 nanograms/milliliter (ng/ml)

The expected normal value for estrogen during the follicular phase is 15–169 picograms/milliliter (pg/ml)

### Statistical methods

The data will be summarized using mean and standard deviation (SD) for the quantitative data and frequency distribution for the qualitative data.

For quantitative data, a comparison between two groups was carried out using a nonparametric *t* test.

The Pearson correlation test is used to estimate the correlation between given random variables. The correlation coefficient indicates the strength and direction of a linear relationship between random variables. Multivariate linear regression analysis was done.

*p* values less than 0.05 were considered statistically significant. All statistical calculations were done using the computer program SPSS (Statistical Package for the Social Sciences; SPSS Inc., Chicago, IL, USA) version 17 for Microsoft Windows.

## Results

Forty patients were included in our study; their age at onset of disease ranged from 16 to 35 years with a mean of 25.38 ± 4.9. The duration of illness ranged from 1 to 12 years with a mean of 4.85 ± 3.11. The number of relapses ranged from 2 to 9 attacks during the duration of illness with a mean of 3.93 ± 1.85. The EDSS ranged from 1 to 5.

### Comparative results

The levels of estrogen, testosterone, interleukin 10, interleukin 4, and tumor necrosis factor alpha (TNF-α) were higher in patients compared to control, but the difference was not statistically significant as illustrated in Table 1.

**Table 1:** Comparison between patients and control subjects as regards hormonal profile and cytokine level

	Patients		Control		*p* value
	Mean ± SD	Range	Mean ± SD	Range	
Estrogen level, pg/ml	1.84 ± 0.27	1.29–2.49	1.74 ± 0.2	1.42–2.27	0.12
Testosterone level, ng/ml	0.66 ± 0.34	− 0.6–0	0.58 ± 0.25	− 0.6–0	0.359
IL-10 level, pg/ml	1.3 ± 0.47	0.43–2.37	1.13 ± 0.48	0.36–1.9	0.19
IL-4 level, pg/ml	1.19 ± 0.511	0.30–2.45	1.1 ± 0.15	0–1.26	0.33
TNF-α level, pg/ml	0.24 ± 0.71	0–2.8	0.15 ± 0.52	0–1.18	0.6

N.B. the range documented in the table is after the normalization of the sample (by using the logarithm of 10)

*IL-10* interleukin 10, *IL-4* interleukin 4, *TNF* tumor necrosis factor alpha

### Correlative results

**Fig. 1:**
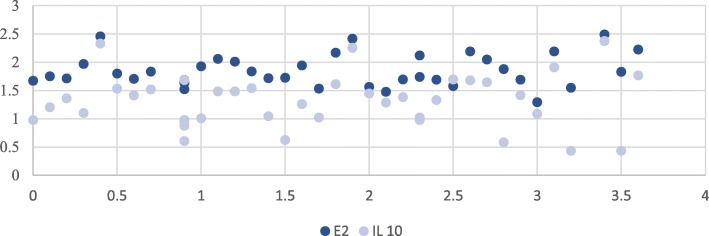
Correlation between estrogen levels and interleukin 10 (IL-10) levels in patients

**Fig. 2:**
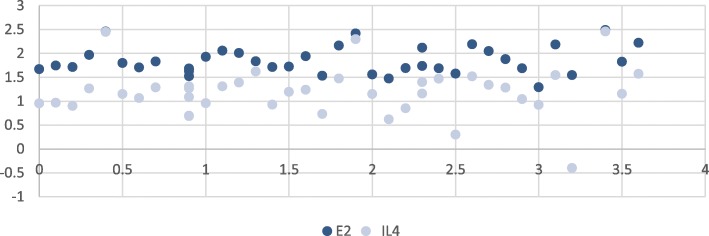
Correlation between estrogen levels and interleukin 4 (IL-4) levels in patients

**Fig. 3:**
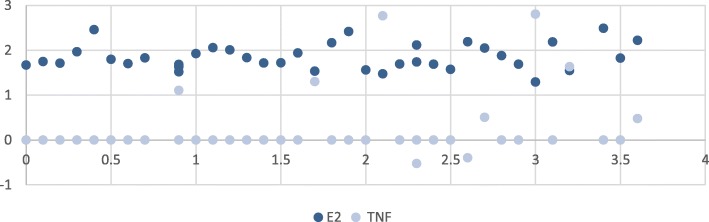
Correlation between estrogen levels and tumor necrosis factor alpha (TNF-α) levels in patients

Based on Pearson’s correlation coefficient, estrogen level showed significant positive correlation to IL-10 (*r* = 0.64, *p* ˂ 0.00001) and IL-4 (*r* = 0.78, *p* ˂ 0.00001) and significant negative correlation to TNF-α levels (*r* = − 0.44, *p* = 0.004), where higher estrogen levels associated with higher IL-10 and IL-4 and lower levels of TNF-α (Figs. 1, 2, and 3), but no significant correlation was found with EDSS (*p* ˃ 0.96) as illustrated in Table 2.

**Table 2:** Correlation between hormonal levels (estrogen and testosterone), cytokine levels, and EDSS in patients

	Estrogen levels		Testosterone levels	
	*R*	*p* value	*R*	*p* value
IL-10 level, pg/ml	0.64	˂ 0.00001*	− 0.02	0.9
IL-4 level, pg/ml	0.78	˂ 0.00001*	− 0.1	0.5
TNF-α level, pg/ml	− 0.44	0.004*	0.08	0.6
EDSS	− 0.007	0.96	− 0.25	0.11

*IL-10* interleukin 10, *IL-4* interleukin 4, *TNF* tumor necrosis factor alpha, *EDSS* Expanded Disability Status Scale

*Significant *p˂*0.05

No statistically significant correlation was found between testosterone, cytokines (IL-10, IL-4, TNF-α), and EDSS (*p* ˃ 0.05) as illustrated in Table 2.

There was no statistically significant correlation between cytokine profile (IL-10, IL-4, TNF-α) and EDSS (*p* ˃ 0.05).

Multivariate linear regression analysis using the cytokine profile (IL-10, IL-4, and TNF-α) as the dependent variable revealed estrogen level as the independent variable (*p* ˂ 0.001, ˂ 0.001, and 0.048, respectively).

## Discussion

Our study confirms the hypothesis that the cooperation between the immune, endocrine, and nervous system could be a possible reason for the influence of sex hormones in MS.

The present study showed that though not reaching statistical significance, estrogen and testosterone levels in the follicular phase were higher in patients compared to control with no significant correlations between sex hormone levels and EDSS score. The results obtained in the present study are in agreement with previous reports.

Zakrzewska-Pniewska and colleagues [7] studied 46 women with a definite diagnosis of MS and 50 healthy women as the control group and determined that 56% of the patients had hormonal disorders, particularly increased levels of estrogen. Furthermore, in a study conducted by Shahdaeizadeh and colleagues [8] on 30 female patients with relapsing-remitting MS (RRMS) and 30 healthy female controls, patients were divided into three groups based on their expanded disability status scale (< 1.5, 1.5–3, and > 3). They reported that the level of estrogen was higher in the follicular phase in all patient groups than in the control group; in addition, it was significantly higher in the patients with EDSS > 3.

Conversely, Trenova and colleagues [9] found that 60% of patients had below the lower limit of normal serum concentrations of estrogen and/or progesterone in one or both phases of RRMS (relapse and remission). Hormonal levels increased significantly during remission in these patients.

Foroughipour and colleagues [10] found that testosterone levels in MS, especially during the active phase of the disease, are decreased, and this reduction was consistent with the severity of brain lesions; in addition, magnetic resonance imaging (MRI) results confirmed these data [5].

Previous reports have demonstrated that testosterone plays an important role in repairing brain lesions and shows protective effects in patients and animal models of MS [10]. In addition, testosterone and its metabolites such as dehydroepiandrosterone (DHEA) directly or through conversion to estrogen by the aromatase enzyme could affect the estrogen receptor [11]. There is evidence that testosterone and estradiol downregulate reactive gliosis and astrocyte proliferation [12] which are the major problems to axonal regeneration in the mammalian CNS [13].

Our results regarding IL-10 and TNF-α revealed that relapsing-remitting MS patients showed no difference in serum levels of proinflammatory (TNF-α) and anti-inflammatory (IL-10) cytokine levels when compared with controls, and this was consistent with previous studies [14–16]. This can be explained by the fact that increased levels of cytokines are associated with the clinical activity in RRMS and the development of a chronic progressive disease. As regards IL-4, our results came contradictory to the previous studies revealing that IL-4 levels were higher in RRMS patients compared with controls [17, 18].

Our results could not confirm the imbalance between Th1 and Th2 cytokines which is an oversimplified explanation for MS pathogenesis and that other cells may play an important role in pathogenesis such as B cells, T helper 17 (Th17), and T reg. A growing importance has been attributed to Th17 cells, a subset of CD4+ effector T cells, in regulating the immune-mediated inflammatory response, and Th17 cells have been indicated as playing a major role in the pathogenesis of several inflammatory and autoimmune disorders. Th17 cells are mainly characterized by the expression of IL-17 which is a proinflammatory cytokine that activates T cells and other immune cells, producing a variety of cytokines, chemokines, and cell adhesion molecules [19].

In the current study, a statistically significant positive correlation was found between estrogen level and levels of interleukin 10 (IL-10) and interleukin 4 (IL-4); additionally, a significant negative correlation was found between estrogen and tumor necrosis factor alpha (TNF-α) (*p* = 0.004).

This conforms with Javadian and colleagues [20] who found that estrogen significantly increased IL-10 and IL-4 and significantly decreased the expression of TNF-α mRNA in peripheral blood mononuclear cells (in vivo) and the culture supernatant of cells (in vitro) stimulated with proteolipid protein (PLP) and mitogen phytohemagglutinin (PHA) in RRMS and control group. Moreover, Soldan and colleagues [21] reported that oral estrogen treatment was associated with significant increase in levels of IL-10 and decreased levels of TNF-α in stimulated peripheral blood mononuclear cells isolated during estrogen treatment.

Correlations found between estrogen and cytokine levels (IL-10, IL-4, TNF-α) were attributed to the anti-inflammatory effect of estrogens that mainly inhibit inflammation and demyelination [22] and causes the downregulation of TNF-α production [23] in addition to the fact that estrogen is capable of modulating both pro- and anti-inflammatory activities of CD4+ T cells and consequently has the ability to influence the outcome of CD4+ T cell-mediated immune responsiveness [24]. This outcome can vary depending upon the level of estrogens, cell type, activation state of cells, local environment, and the experimental context, and the estrogen-mediated effects are apparent in all major innate and adaptive immune cells; also, natural varied expression of estrogen receptors in different tissues and cell types balances the overall outcome of estrogen-mediated immune responses [25]. So, estrogen at high concentrations has a stimulatory effect on the anti-inflammatory pathway [26] and shifting cytokine levels towards Th2 immunity [27].

## Conclusions

We concluded that sex hormones have an influence on the immune system; besides, estrogen has an anti-inflammatory effect on cytokine milieu, and therefore, it can be tried as a treatment option in multiple sclerosis.

## Abbreviation

APCs: Antigen-presenting cells
CD: Cluster of differentiation
CNS: Central nervous system
DHEA: Dehydroepiandrosterone
EDSS: Expanded Disability Status Scale
ELISA: Enzyme-linked immunosorbent assay
HRP: Horseradish peroxidase
IL-10: Interleukin 10
IL-4: Interleukin 4
MRI: Magnetic resonance imaging
MS: Multiple sclerosis
PHA: Mitogen phytohemagglutinin
PLP: Proteolipid protein
RRMS: Relapsing-remitting multiple sclerosis
T reg: T regulatory
Th1: T helper 1
Th2: T helper 2
TNF-α: Tumor necrosis factor alpha
